# The Value of Differential Diagnosis in Dentistry

**Published:** 1896-07

**Authors:** V. A. Latham

**Affiliations:** Chicago


### THE DENTAL REGISTER.

VOL. L.] JULY, 1896. [No. 7.

### COMMUNICATIONS.

### The Value of Differential Diagnosis in Dentistry.

BY V. A. LATHAM, M.D., CHICAGO.

Read before the Section on Dental and Oral Surgery at the forty-sixth annual meeting of the American Medical Association, at Baltimore, Md., May 7th to 10th, 1895.

The subject of diagnosis is one of much importance to a practitioner, and the man who can diagnose rapidly and accurately is usually successful in his profession. In medical works great importance is laid on diagnosis, even to such a degree that special works are written on the subject. Who does not remember the differential tables in nervous, respiratory and cardiac diseases, and the dread they inspired at examinations? Without their aid we are not able to diagnose our cases, and hence the urgency of requiring students to learn them. I have constantly been surprised at the difficulties which students encounter in dental surgery and pathology. Even a well-read student finds it hard to bring his knowledge to bear promptly and efficiently upon the patient before him. The recognition of the several symptoms which the student has learned from lectures or reading can be best directed by the teacher at the side of the operating chair, just as it is at the bedside in medicine; but in his absence it is not always easy for the student to get a clue to the nature of the case before him. Every member of the dental profession can recall the days of his service in the operating room, and remember his difficulty in procuring help from the professor or his assistant, for as a rule, the corps of instructors in our colleges, medical and dental, is much too small to allow more than a passing glance or word to an inquiring student, and he does the best he can. Whether this is a wise plan or not is a subject for discussion, for several reasons. First and foremost, the student is incapable, in the majority of cases, of understanding the treatment of his earlier cases. Again, the patient, even a charity case, under these conditions is entitled to good judgment in his diagnosis, though willing to submit to the student operator; his position demands the supervision of a chief, not only for the care and result of the operation, but for the welfare and regard of the institution in which the operation is held. It is not always a patient's fault that he is a public case, and indeed, we, as a class of students, graduates or undergraduates, must remember it is through their misfortune we obtain our practical experience, and so ought to give every care and attention to do the best we can for the "subjects of the clinics." The larger the clinics, the more benefit we may derive from them. The larger the corps of capable demonstrators under the guidance of an experienced diagnostician as "chief," the more practical and successful professional men and women we become.

It is evident that before any one can successfully treat a disease, he must be acquainted with its nature and the symptoms it produces. For instance, before prescribing for a patient suffering from pain in the head, he must ascertain from what that pain arises. It may result from a carious tooth, when treatment or extraction will give relief; or from periostitis, when potassium iodide will be beneficial; or from constipation, for which only a purgative is required; or again, it may be symptomatic of an incurable disease of the brain, which might be aggravated by many of the remedies well fitted for the cure of a less formidable disorder.

Diagnosis is the science which teaches us thus to distinguish one disease from another, and to trace symptoms to the causes from which they spring. Now, diagnosis is valuable not only for treatment, but it enables you to form an accurate opinion as to the future course of a disease. For example, two persons may complain of palpitation of the heart; in the one the organ is healthy in its structure, but unduly excited by disordered digestion; in the other it may be affected with an incurable disease that may at any moment terminate the patient's life. It seems a curious fact that works on dental surgery are so very imperfect and rambling on the subject of diagnosis. I doubt if any branch of medicine is so poor in this respect. What is the reason? I suspect it lies in five sources:

1. The hurry to obtain a diploma.
2. The study of *only just* what seems absolutely necessary in the practice of dentistry, and a corresponding inability to apply general principles.
3. The fear of encroaching on general medicine.
4. Insufficient preliminary *education.*
5. Lack of a thorongh knowledge of the *normal conditions;* and a habit of relying too much upon one mode of treatment.

Diseases are distinguished from each other by such alterations in the organs themselves, or in their secretions, as can be ascertained by the senses of the observer (physical signs); or by changes in the functions of the parts affected (symptoms). The physical signs are least liable to misconception, inasmuch as we are independent of any suggestion on the part of the patient. Thus we see a carious tooth, or an exposed pulp, or a swelling, we know that there must be some abnormal condition of the part. Physical signs can not be exclusively relied upon for the formation of a diagnosis; the symptoms and history of the case must also be taken into consideration. It is generally difficult for a student to guide a patient's account of his complaint in such a way as to derive the necessary information from it. Most persons ramble in describing symptoms, and insist, in many cases, on giving their own or other persons' opinions as to the nature of their disease, instead of confining themselves to a narration of facts. The only way to overcome this is by conducting your examination in a systematic manner, and by having a definite aim in every question you ask. Students are apt to suppose that some particular sign or symptom is sufficient to indicate each disease. Unfortunately this is not the case. On the contrary, we can rarely diagnose any morbid condition without taking into consideration a number of symptoms; indeed, we are often forced to determine the nature of a malady by proving what it is *not* rather than what it is—diagnosis by exclusion.

The subject of "taking cases" ought to be insisted upon in all medical and dental hospitals and colleges, and all students should be required to examine cases and to write out their histories in their operating or clinic service, the reports to be read and criticized before the class by the professors of the several subjects. To know which points are the important ones is anything but easy, and can only be acquired by experience and through sound teaching. How many valuable cases become wholly worthless from the reason of bad or incomplete histories is only known to those in the profession who try to do legitimate and scientific study. Hospital internes are generally poor history takers because they are badly trained, and are often overwhelmed with work, the number of patients allotted to each interne being too great to permit thorough, painstaking work. This is often injustice to the patients, and always so to the interne; for the value of his appointment lies in the opportunity for the close study of his cases, under a staff of skilled consultants, from whom he can inquire concerning obscure points in signs and symptoms, and whose discussions and remarks upon treatment and results serve to impress the points indelibly upon his memory. For this training he gives his time and strength, and certainly has a right to all the privileges of his position; he ought not to be worked like a machine! It is not the number of gold fillings inserted in a day, but the nature of the caries, its etiology, treatment and results we are after. What good is it to teach a student to insert a gold filling with a perfect adaptation and contour if he does not recognize the variety of decay, the resistance and pathologic conditions offered by the histologic elements, the condition of the gums, the development and articulation of the maxillæ and teeth, the state of the secretions of the stomach and oral cavity, as well as the general condition of the patient? It is worse than useless to expect good results, except by chance, without accurate knowledge on these vital points. For we must remember that the general health will permit only of temporary measures in such constitutional states as diabetes, locomotor ataxia, pregnancy and in convalescence from the acute fevers.

The most difficult problems, perhaps, we have to diagnose in dental pathology are the neurologic. Dental irritation is one of the commonest and most powerful causes of reflex nervous disturbances. We all know of many cases treated by the medical profession for months which might have been relieved by attention to the teeth. Understand, I do not mean to omit the medical treatment of such diseases as syphilis, malaria, neoplasms and so forth. The terminations of the fifth nerve, namely the pulps, seldom allow increased circulation because of the resistant walls surrounding them, by which the entire pressure is reflected back to the central nervous system and to all peripheral nerves. Even when the sufferer indicates a special tooth we may find the trouble in the other jaw and on the opposite side. It may not be amiss to consider here some varieties of pain and their indications. Two sources of dental pain are: 1, the nerves of the tooth-pulp; the dentinal fibrils and the contents of the interglobular spaces of dentine; 2, the alveolo-dental periosteum.

Pain arising from the pulp and from the dentinal fibrils is always the same in character, but may vary in degree. The pulp and protoplasmic elements of a tooth may set up a localized pain; but usually the pain arising from these structures is now localized, being felt at a distance. Periosteal disease, on the contrary, invariably causes local pain, and never pain at a distance, or only such as is quite insignificant when compared to local pain. Thus, true neuralgia never arises from uncomplicated alveolo-dental periostitis. Neuralgia, therefore, never arises uncomplicated from a dead tooth. This, as proven by experience, helps us to arrive at a correct diagnosis of the cause of one variety of dental pain. Often obstinate cases of obscure neuralgic pain can be cured by curing alveolo-dental periostitis of a dead tooth. This may be explained by assuming that the periostitis is complicated by some irritation or inflammation of a neighboring pulp; which irritation or inflammation may be passed on from the inflamed periosteum to the pulps in its proximity by continuity of tissue. When this is the case the pulp is attacked from the proximal end, the inflammation following the apical foramenal nerve and vessels. Where alveolo-dental periostitis occurs in live teeth there is almost always some irritation or inflammation of the pulp, which may be secondary or otherwise. The following are modes of diagnosticating causes of pain in or from the teeth :

1. Explore with the mirror and a sharp-pointed probe ; find the cavities, then test for living or dead pulps. If the deep cavities are sensitive, or the nerves exposed, here is the probable cause of the pain. These are our easy, straightforward cases. If, however, no pulp canals are open, then we must—

2. Test by percussion and thermal changes. If the pulp is found unduly sensitive, hyperemia and irritation are indicated; if insensitive to thermal changes, partial or complete necrosis may be diagnosed. (The heat test must be applied for some time, so the tooth feels hot to the finger, before necrosis can be regarded as proven.) This is not always true, though it is fair to conclude that hypersensitiveness to thermal changes indicates the tooth as a probable cause of neuralgia, while diminished sensibility is as often a sign of partial or complete necrosis. If we are still doubtful—

3. Drilling is justifiable, for if the pulp be alive, though much receded by calcification, pain will generally be felt by the cutting of the dentinal fibrils before the pulp is reached. Carefully distinguish, however, between pain caused by the pressure of the instrument in cases where alveolo-dental periostitis is present, and that due to hyperæmic pulp. Any branches of the fifth nerve in the head, face or teeth, or even more remote nerves, as the superficial cervical, or even the brachial nerves, may be the seat of neuralgic pain arising from any tooth on that side. The pain, however, never crosses the vertical median line, often the faulty teeth being unsuspected by the patient. Reflex neuralgia from such a tooth may occur anywhere, but usually its favorite location is where the nerves emerge from foramina and where they anastomose. There are also certain preferred sites for certain teeth. For example, pain in the ear and pain shooting down the shoulder and into the arm, if dental in origin, is almost certain to arise from an inferior tooth. Pain, supra and infra-orbital, almost always arises from a superior tooth. Temporal pain, pain in front of tragus over auriculo-temporal nerve, or parietal pain, "parietal focal spot," usually arises from a tooth in either mandible. Pain felt in one tooth is often due to a companion tooth in the other mandible. Pain widely diffused in character, *i. e.*, shooting along many branches of the fifth nerve at one and the same time, is usually from a tooth far back in the mouth, third molar. Pain of a live pulp is not continuous but capricious, lasting a short time and suddenly disappearing, periodic in appearance, frequently benefited by medicines, as salicylate of soda, quinine and other tonics or stimulants. In both these particulars neuralgia arising from the pulp agrees with neuralgia arising from unknown or constitutional causes (idiopathic neuralgia). This similarity of behavior has often led to a false diagnosis of the etiology of neuralgia on the part of physicians and dentists. The pathologic pulp which causes neuralgia of the usual capricious character is often in a state of chronic inflammation ; acute pulpitis seldom reaching the dental surgeon in the early stage, the pulp soon dies. In pulpitis, cold alone is acceptable to the patient for giving relief, periosteal symptoms being nearly always present. Nerve exhaustion means increased nervous irritability. Anæmia of young subjects, especially, causes severe neuralgia to arise from such slight causes as exposure of dentine at the necks of the teeth, through irritation of the dentinal fibrils and their canalicular terminations.

With regard to etiology of dental periostitis and pulpitis, the diplococcus pneumoniæ may be regarded as a factor, usually accompanied by the staphylococcus pyogenes aureus and albus. Long-continued pulpitis may cause inflammation of the antrum just as well as a severe catarrhal inflammation can do so. Non-eruption of the teeth, which is very hard to diagnose in the early stages, can also cause a purulent condition of the antrum, and may give frequent and persistent neuralgia, as well as trismus from irritation of the inferior dental nerve.

Causes of neuralgic pains are: 1, sensitive dentine; 2, fibroid pulps; 3, crowding; 4, necrosis of pulp in a confined space; 5, exostosis; 6, alveolar periostitis; 7, filling over exposed pulp; 8, malpresentation of third molar; 9, rheumatic and gouty diathesis; 10, anæmic and chlorotic states; 11, serous calculus on roots; 12, malaria; 13, pulp nodules; 14, sympathy; 15, recession and absorption of gum and alveolus; 16, pressure of gases in the pulp chamber; 17, traumatism.

The most pernicious form of neuralgia is that which is due to septic influences occurring after some traumatic injury, in which paresis or paralysis of the nerve effected sometimes follows.

Differential Diagnosis between Antral Abscess and Ozena.—Points in favor of the former are: 1, the presence of pulpless teeth; 2, a shortening of the face from the oral cavity to the orbit; 3, accumulation and sudden reaccumulation of pus, showing at the hiatus, in middle meatus, half an inch from anterior extremity of inferior turbinated bone; 4, discharge of pus increased on putting patient in horizontal position, especially on the sound side; 5, relative darkness over the diseased maxilla when the bones of the face are illuminated; 6, puncture through nose and aspiration of fluid; 7, presence of carious teeth, especially roots, in upper màndible. Bulging of the cheek and deformity of the jaw are not always present. Ozena is recognized by: 1, characteristic fetid discharge; 2, olfactory anæsthesia; 3, detection of denuded necrosed bone in nares; 4, presence of crusts of dried secretions and ulcers, especially in nasopharynx; 5, the teeth may be normal; 6, diathesis (syphilitic or strumous).

The following appended tables may be useful:

| *Sensitive Dentine.* | *If the Pulp is Sensitive.* |
|---|---|
| When the examination over a considerable part of the cavity walls does not respond to simple pressure. Pain is not persistent. | When the examination is made near the pulp it responds to pressure. |

*Hyperœmia of the Pulp.*

Pain of boring character; tooth highly sensitive to hot and cold temperatures, and painful in mastication and on pressure. Hard to distinguish from exposed dentine. Absence of throbbing pain (pathognomonic of pulpitis); serious neuralgia may be a symptom.

*Pulpitis.*

Pain of a boring character rapidly increasing, assuming a throbbing form, extending from the diseased tooth to the neighboring teeth and to the side of the face, the tooth forming the center of its intensity. The larger and younger the pulp the greater the pain. In time the pain subsides, to return, though, on the slightest provocation, or the horizontal position being resumed. Pulp injected with blood throughout, the exposed part deeper in color.

*Chronic Pulpitis.*

Pain less severe than in the acute form, not very intense nor long in duration when present. Comes on at irregular intervals —often vague neuralgic pains. Sudden changes of temperature, or applications of irritants will produce a paroxysm of pain, lasting from a few minutes to hours. Pulp shows inflammation limited to exposed spots, the rest pale and healthy.

*Necrosis of Pulp.*

Pain becomes changed to a dull, heavy ache with feeling of tension. Tooth feels too long, raised in alveolus by the pericementitis and periostitis, and loosened; may have some swelling at the side, on the gum or at the root; a change of color can be seen by a strong light, the dead tooth having a dark line in the pulp region.

It is often difficult to distinguish pulpitis from hyperæmia. In hyperæmia the throbbing character of the pain is not so well marked and the pulp usually not exposed. Pulpitis must also be distinguished from periostitis; the differential points being as follows:

*Pulpitis.*

Pain, sharp, lancinating or throbbing, intermittent and reflected within the tooth.

Thermal changes cause pain.

Pressure or percussion on tooth gives no pain at first.

Slight pressure on a piece of wool in cavity generally gives acute pain.

With pulpitis we may have pericementitis by continuity.

*Periodontitis.*

Pain, dull, heavy and constant, and without the tooth.

Thermal changes do not cause pain.

Pressure or percussion on tooth gives pain from the first.

Slight pressure does not give pain except through pressure transmitted to the periosteum.

Tooth loosens and elongates.

A student may be well-versed in the sciences of his profession, and yet unable to diagnose the simplest case of disease presented in actual practice, simply because the knowledge has not become his own in sufficient degree to enable him to comprehend appearances. Diagnosis, to be useful, must not only be clear and satisfactory to our own minds, but made so plain to those in charge of the case as to render them alive to the real necessities present, thus securing that co-operation without which treatment will be ineffectual, if not actually a damage to those we essay to benefit. No diagnostician who is conscientious as well as competent will set up his own opinion as the standard, but will always rely upon the perception of those to whom he wishes to make the diagnosis plain, and in this very idea is the safety, for the recapitulation of the case may reveal points previously unnoticed, so giving certainty and confidence to the patient and himself. To become a good diagnostician it is imperative to understand principles and laws in preference to formularies and modes, and to comprehend these one's powers must not only be good, but kept in constant use, thus insuring the best and latest methods, reading and experience.

